# Curcumin attenuates prostatic hyperplasia caused by inflammation *via* up-regulation of bone morphogenetic protein and activin membrane-bound inhibitor

**DOI:** 10.1080/13880209.2021.1953539

**Published:** 2021-08-06

**Authors:** Yuhang Liu, Zhaohui Wang, Yu Gan, Xiang Chen, Bo Zhang, Zhi Chen, Peihuan Liu, Bingsheng Li, Feng Ru, Yao He

**Affiliations:** a Department of Urology, Hunan Children's Hospital, Changsha, Hunan, China; b Department of Urology, Xiangya Hospital, Central South University, Changsha, Hunan, China

**Keywords:** Benign prostatic hyperplasia, epithelial-mesenchymal transition, lipopolysaccharide, Toll-like receptor 4, tumour necrosis factor-α

## Abstract

**Context:**

Inflammation and epithelial-mesenchymal transition (EMT) play important roles in the occurrence and development of benign prostatic hyperplasia (BPH); curcumin exerts anti-proliferative, anti-inflammatory, and anti-EMT effects.

**Objective:**

To explore the anti-inflammatory and anti-EMT mechanisms of curcumin in BPH.

**Materials and methods:**

Ten-week-old male C57BL/6 mice were administered lipopolysaccharide (LPS, 100 µg/kg) in the prostate lobules to establish an inflammatory BPH model (LPS group), and curcumin (120 mg/kg) was administered into the abdominal cavity for 2 weeks (three times a week, curcumin-treated group). A group of healthy mice served as the control group. The expression of Toll-like receptor 4 (TLR4), bone morphogenetic protein and activin membrane-bound inhibitor (BAMBI), EMT markers, inflammatory cytokines, and transforming growth factor β1 (TGF-β1) was detected by PCR and western blotting. TGF-β1 (0.1 ng/mL) and LPS (100 ng/mL) were used to induce EMT in benign prostatic hyperplasia epithelial cells (BPH-1).

**Results:**

*In vivo*, curcumin reduced the size of the prostate, suppressed the expression of vimentin and TLR4, and increased the expression of E-cadherin and BAMBI in the LPS-induced BPH mouse model. Moreover, curcumin decreased the levels of IL-6 and TNF-α by 44.52 and 46.17%, respectively. *In vitro*, curcumin attenuated cell proliferation, suppressed the expression of vimentin and TLR4, and increased the expression of E-cadherin and BAMBI in BPH-1 cells. Furthermore, BAMBI knockdown reversed the expression of vimentin and E-cadherin induced by curcumin.

**Discussion and conclusion:**

This study demonstrated that curcumin alleviated hyperplasia, EMT, and inflammation *in vivo*. Furthermore, curcumin suppressed EMT by targeting BAMBI via the TLR4/BAMBI/TGF-β1 signalling pathway *in vitro*, demonstrating its potential utility in BPH treatment.

## Introduction

Benign prostatic hyperplasia (BPH) affects men, and its incidence increases with advancing age (Madersbacher et al. 2019). Studies have shown that more than 50% of men older than 50 years have BPH (Chughtai et al. 2016). Patients with BPH often have noticeable lower urinary tract symptoms (LUTS) resulting from changes in the anatomy of the lower urinary tract (Abrams et al. 2002; McVary et al. 2011). In addition, a previous study demonstrated that patients with BPH and chronic prostatitis have more severe LUTS and a high risk of urinary retention (Nickel et al. 2016). It not only seriously affects the quality of life of patients, but also increases the economic burden on society (Ventura et al. 2011; Speakman et al. 2015). Sex hormone levels, inflammation, and age are considered to be closely related to the pathogenesis of BPH (Gandaglia et al. 2013; Chughtai et al. 2016; De et al. 2016). Prostatic inflammation plays a crucial role in the pathogenesis and progression of BPH (Akanni et al. 2020). A study by Robert et al. (2009) conducted on 282 patients who underwent BPH surgery reported that the International Prostate Symptom Score (IPSS) and prostate volume had a significant correlation with prostatic inflammation. They also reported that chronic prostatitis is associated with 79, 48, and 20% of patients with severe, moderate, and no BPH, respectively. In addition, the significant correlation between BPH and chronic prostatitis was further verified by an autopsy that was conducted on Asian and Caucasian men (Zlotta et al. 2014). After investigating the occurrence and development of BPH, Vignozzi et al. (2014) proposed a three-hit hypothesis of infection, metabolic changes, and hormonal disorders, and indicated that long-term inflammation induces excessive production of growth factors, which consequently induces prostate remodelling and furthers prostate enlargement (Vignozzi et al. 2014). This evidence indicates that inflammation is an important factor in the development of BPH. At present, frequently used therapeutic drugs, including alpha-blockers and 5-α reductase inhibitors, are not completely effective in certain patients (Ventura et al. 2011; Bechis et al. 2014). Besides, anti-inflammatory drugs are not included in the list of clinical drugs for BPH treatment (Gupta et al. 2015). Furthermore, non-steroidal anti-inflammatory drugs commonly used in clinics have side effects; thus, these drugs are not suitable for long-term use in patients with BPH (Geusens et al. 2013).

Currently, specific natural plants and components with anti-inflammatory effects, such as asters, rhubarb, and protocatechuic acid, epigallocatechin-3-gallate have shown promising potential in the treatment of BPH (Zhou et al. 2018; Akanni et al. 2020; Rho et al. 2020). Curcumin (Cur), a plant extract that has clinical application in prostatic inflammation (Cosentino et al. 2016), as well as anti-fibrotic effects (Patel et al. 2020), has been demonstrated to be safe, with the potential to treat BPH (Kim et al. 2015); however, its molecular mechanism is not yet clear.

Being a receptor of Toll-like receptors (TLR) 4, lipopolysaccharide (LPS) can downregulate the pseudo-receptor of TGF-β1 [bone morphogenetic protein and activin membrane-bound inhibitor (BAMBI)], resulting in activation of the transforming growth factor (TGF)-β1/Smad signalling pathway and mediating the epithelial-mesenchymal transition (EMT) of prostatic hyperplasia cells (He et al. 2016). It is suggested that BAMBI may play a ‘hub’ role between the LPS/TLR4 signalling pathway and the TGF-β1/Smad signalling pathways (He et al. 2016).

In the present study, we demonstrated that curcumin alleviated hyperplasia, EMT, and inflammation *in vivo*. Moreover, curcumin suppressed EMT by targeting BAMBI through the TLR4/BAMBI/TGF-β1 signalling pathway *in vitro*.

## Materials and methods

### Drug

Curcumin (purity > 98%, 08511-10MG) was purchased from Sigma (Chemical Co., St. Louis, MO, USA), and lipopolysaccharide (purity ≥ 99%, L8880-10 mg) was purchased from Solarbio (Beijing, China).

### Animals and treatments

Twenty-four 10-week-old wild-type C57BL/6 male mice were used for *in vivo* experiments and obtained from the Department of Experimental Animals, Central South University, China. The mice were housed in a specific pathogen-free animal facility and the temperature in the facility was maintained at 22**–**24 °C with an alternating 12 h light/dark cycle (8:00 am and 8:00 pm). All mice were given free access to food and water. The experimental planning and procedures were approved by the Science Research Centre of Central South University (No. 2019sydw0149) Changsha, China. All experiments were conducted by the guidelines of the National Institutes of Health. Mice were randomly divided into the following three groups: (1) PBS (phosphate buffer saline) group (control group) (*n* = 8), (2) LPS group (*n* = 8), and (3) LPS + curcumin group (curcumin***-***treated group) (*n* = 8). The mice were anaesthetized with pentobarbital sodium (80 mg/kg), and their lower abdomen was then cut to expose the prostate. Subsequently, PBS (100 µL) was injected into the prostate lobes of mice in the control group, while LPS (100 µg/kg) was injected into the prostate lobes of mice in the LPS and LPS + curcumin groups. Subsequently, the wound was disinfected and sutured.

Curcumin was dissolved in the cell cryopreservation solution (dimethyl sulfoxide (DMSO), and olive oil was then added to obtain an emulsion with the concentration of 4.8 mg/mL. The emulsion (0.025 mL/g) was injected into the abdominal cavity of the mice in the LPS + curcumin group (three times a week), while olive oil (0.025 mL/g) was injected into the abdominal cavity of the mice in the PBS and LPS groups at the same frequency. After 14 days, all mice were anaesthetized with pentobarbital sodium. The prostate lobes were carefully separated and washed with ice-cold PBS. The tissue was then fixed with 4% paraformaldehyde solution and stored at −80 °C for further experiments.

### Cell culture and stimulation

Benign prostatic hyperplasia epithelial cell line (BPH-1) was acquired from the American Type Culture Collection (ATCC) and cultured in RPMI 1640 (Sigma, USA) supplemented with 1% penicillin, 1% streptomycin, and 15% foetal bovine serum (Gibco, China). The cell stimulation experiment consisted of 5 groups: (1) blank control group (control group), (2) LPS group, (3) TGF-β1 group, (4) LPS + TGF-β1 group, and (5) LPS + TGF-β1 + Cur group (experimental group). Cells were stimulated with either 100 ng/mL LPS with or without 0.1 ng/mL TGF-β1 (Santa Cruz Biotechnology, USA) or with or without 40 µmol/L Cur.

### Cell viability assay

Cells (1 × 10^4^ cells/well) were seeded in 96-well plates. The viability of BPH-1 cells receiving various treatments was detected using a Cell Counting Kit 8 (CCK-8, Donghuan Biotech, Shanghai, China).

### Histopathology

Prostate tissue was dissected, fixed with 4% paraformaldehyde solution, and embedded in paraffin to prepare tissue sections for histopathological and immunohistochemical analysis. Subsequently, these prostate sections were stained with haematoxylin and eosin (H&E) and Masson staining, as previously described by Zhang et al. (2019).

### Immunohistochemistry and fluorescence immunohistochemistry analysis

For immunohistochemical staining of prostate specimens, paraffin sections were deparaffinized and rehydrated. The sections were incubated with 3% hydrogen peroxide solution for **∼**12 min and then incubated in a blocking solution containing 5% bovine serum albumin for 30 min. Next, the sections were incubated with anti-BAMBI (1:100; Cell Signalling Technology; USA) and anti-TLR4 (1:100; Novus; USA) antibodies at 4 °C overnight, followed by incubation with horseradish peroxidase (HRP). The secondary antibody (1:100; Sigma-Aldrich, USA) was incubated at 25**–**28 °C. Images were obtained using a microscope (Nikon, Tokyo, Japan).

BPH-1 cells were fixed with 4% paraformaldehyde at room temperature for 30 min. After washing with PBS, the cells were permeabilized with 0.5% Triton X-100 for 15 min. Briefly, the endogenous peroxidase activity of the cells was blocked by treatment with 3% H_2_O_2_/PBS for 20 min. They were subsequently blocked with 0.2% fish gelatine for 20 min and incubated with primary antibodies, anti-TLR4 (1:100; Novus, USA), and anti-BAMBI (1:100; Cell Signalling Technology; USA), overnight at 4 °C. After washing, the cells were incubated with secondary antibodies for 60 min and nuclear staining was performed. Images were acquired using a fluorescence microscope (Nikon, Tokyo, Japan).

### Isolation of primary prostate epithelial cells

Mouse prostate was cut into fragments (∼1-mm^3^) after washing with PBS and the tissues were digested using 1% collagenase (three steps, each for 30 min), followed by digestion with 1% trypsin (three steps, each for 30 min). Cell suspensions were then washed three times with PBS, followed by centrifugation for cell collection. Single-cell suspensions were prepared after filtering the collected slurry using a 40 μm filter (Merck Millipore Ltd, Co Cork, Ireland). Subsequently, all cells were seeded on polypropylene tissue culture dishes for 12 h that were sufficient for stromal cells to attach, but not for epithelial cells (remained unattached). The supernatant (prostate primary epithelial cells) was collected and used for further experiments.

### Quantitative polymerase chain reaction

We analyzed and measured the mRNA expression levels of BAMBI, TLR4, TNF-α, IL-6, TGF-β1, E-cadherin, and vimentin in mouse prostate tissue (Table 1). Similarly, BAMBI, TLR4, E-cadherin, and vimentin expression levels were measured in BPH-1 cells (Table 2). The detailed steps of the experiment are described in a previous study (He et al. 2016).

**Table 1.** Primer sequence of real-time PCR of mouse prostate tissue.

Target gene	Sequence
GAPDH	
Forward primer	5′-GAAGGTGAAGGTCGGAGTCA-3′
Reverse primer	5′-GAAGATGGTGATGGGATTTC-3′
TLR4	
Forward primer	5′-TTTGCTGGGGCTCATTCACT-3′
Reverse primer	5′-CTCGGCACTTAGCACTGTCA-3′
BAMBI	
Forward primer	5′-AAGCAGAATGCAGGGTCTCC-3′
Reverse primer	5′-CCCCCTATGGTGCAGTGTTT-3′
TNF-α	
Forward primer	5′-AGCCGATGGGTTGTACCTTG-3′
Reverse primer	5′-CTCCAAAGTAGACCTGCCCG-3′
IL-6	
Forward primer	5′-GCCCACCAAGAACGATAGTCA-3′
Reverse primer	5′-ACTGGATGGAAGTCTCTTGC-3′
TGF-β1	
Forward primer	5′-TTGCTTCAGCTCCACAGAGA-3′
Reverse primer	5′-TGGTTGTAGAGGGCAAGGAC-3′
E-cadherin	
Forward primer	5′-CTCCACCTCCACCAATGACC-3′
Reverse primer	5′-AGTCAGTATTTCTCAAAGTGGAACA-3′
Vimentin	
Forward primer	5′-CCGGGTCTGCAAGAGTTTCT-3′
Reverse primer	5′-GGATCGCGGGAAGGATTCAT-3′

**Table 2.** Primer sequence of real-time PCR of BPH-1.

Target gene	Sequence
GAPDH	
Forward primer	5′-GAAGGTGAAGGTCGGAGTCA-3′
Reverse primer	5′-GAAGATGGTGATGGGATTTC-3′
TLR4	
Forward primer	5′-ATGTCTTTTTATTCCTGTAGGTGTG-3′
Reverse primer	5′-ACCCGCAAGTCTGTGCAATA-3′
BAMBI	
Forward primer	5′-TCCCGTTTGCACTACAGCTT-3′
Reverse primer	5′-GGCACCATGCATTCCAAGTC-3′
E-cadherin	
Forward primer	5′-GCCCTTTCTGATCCCAGGTC-3′
Reverse primer	5′-TAGCCTGGAGTTGCTAGGGT-3′
Vimentin	
Forward primer	5′-GGACCCTCTTTCCTAACGGG-3′
Reverse primer	5′-TAGTTGGCGAAGCGGTCATT-3′

### Western blot and co-immunoprecipitation assay

Proteins from mouse prostate tissue or BPH-1 cells were extracted from the cell lysate (Sigma, USA) supplemented with protease inhibitors (Abcam, China). The protein concentration was measured using a microplate reader and a bicinchoninic acid (BCA) kit (Beyotime, China). Proteins were separated using SDS-PAGE and then transferred to a nitrocellulose membrane. After blocking with 5% skim milk, the membrane was incubated with the primary antibody for 8**–**12 h at 4 °C, followed by incubation with the secondary antibody for 1 h at 37 °C. A chemiluminescence reagent kit (Advanstar, USA) was used to visualize the proteins.

Co-immunoprecipitation (Co-IP) assay was performed according to the manufacturer’s instructions. Briefly, antibodies against TGF-β1 or BAMBI were added to the resin for 2 h at room temperature for immobilization. The cells were then lysed, and the supernatant was incubated with antibody-immobilized resin at 4 °C for 1 h. The Co-IP fraction was eluted, followed by SDS-PAGE and WB analysis using anti-TGF-β1 or anti-BAMBI as primary antibodies and anti-mouse IgG-alkaline phosphatase antibody (Sigma, USA) as the secondary antibody.

### Statistical analysis

Statistical analysis was performed using the GraphPad Prism software (version 6.0). All results are expressed as mean ± standard deviation (SD). Comparisons among various groups were made by one-way analysis of variance, followed by the Student–Newman–Keuls (S–N–K) method. Statistical significance was set at *p* < 0.05.

## Results

### Effect of curcumin on hyperplasia and inflammation in the LPS-induced BPH mouse model

**Figure 1.**
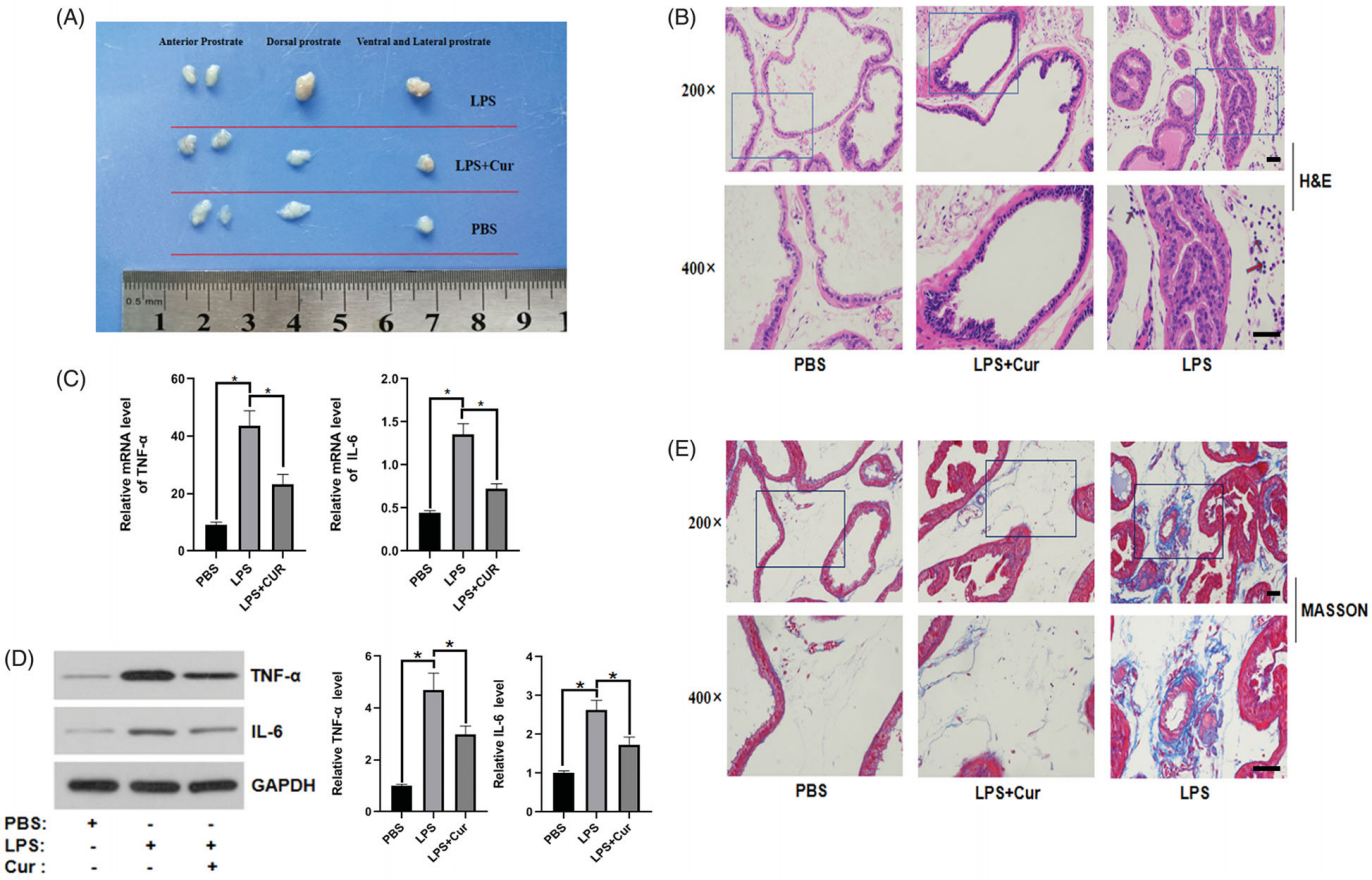
The effect of curcumin on prostate size and expression of inflammatory cytokines in mice. (A) Representative images of the size of mouse prostate in each group. (B) Representative images of different tissues stained with H&E (top panel: 200× magnification, bar = 50 μm; bottom panel: 400× magnification, bar = 50 μm). (C,D) PCR and western blotting analyses of TNF-α and IL-6 in primary mice prostate epithelial cells. *p < 0.05. (E) Representative images of different tissues dyed with Masson's trichrome staining (top panel: 200× magnification, bar = 50 μm; bottom panel: 400× magnification, bar = 50 μm).

As previously reported by Xu et al. (2019), a BPH mouse model was established after intraprostatic injection of LPS. As shown in Figure 1(A), the size of the mouse prostate in the LPS group was notably larger than that in the control group. Treatment with curcumin reduced the size of the prostate, which was induced by LPS. H&E staining revealed additional basal membrane folding, a higher number of acinar papilliform projections, wider lumen space, thicker epithelium, and tighter stroma in the LPS group than in the control group. However, the morphological changes induced by LPS were significantly improved following curcumin treatment (Figure 1(B)). Masson’s trichrome staining revealed improvement in the deposition of extracellular matrix (ECM) collagen in the LPS group compared to that in the control group, while the administration of curcumin decreased the enhanced deposition induced by LPS (Figure 1(E)). Curcumin has been known to downregulate the expression of inflammatory cytokines (TNF-α and IL-6) (Jain et al. 2009; Das & Vinayak 2014). Therefore, we detected the expression of inflammatory chemokines in prostate tissues by PCR and western blot analyses. The expression of TNF-α and IL-6 was lower in the LPS + Cur group than in the LPS group (Figures 1(C,D)). These results indicate that curcumin alleviated hyperplasia and inflammation in the LPS-induced BPH mouse model.

### Effect of curcumin on EMT in the LPS-induced BPH mouse model

**Figure 2.**
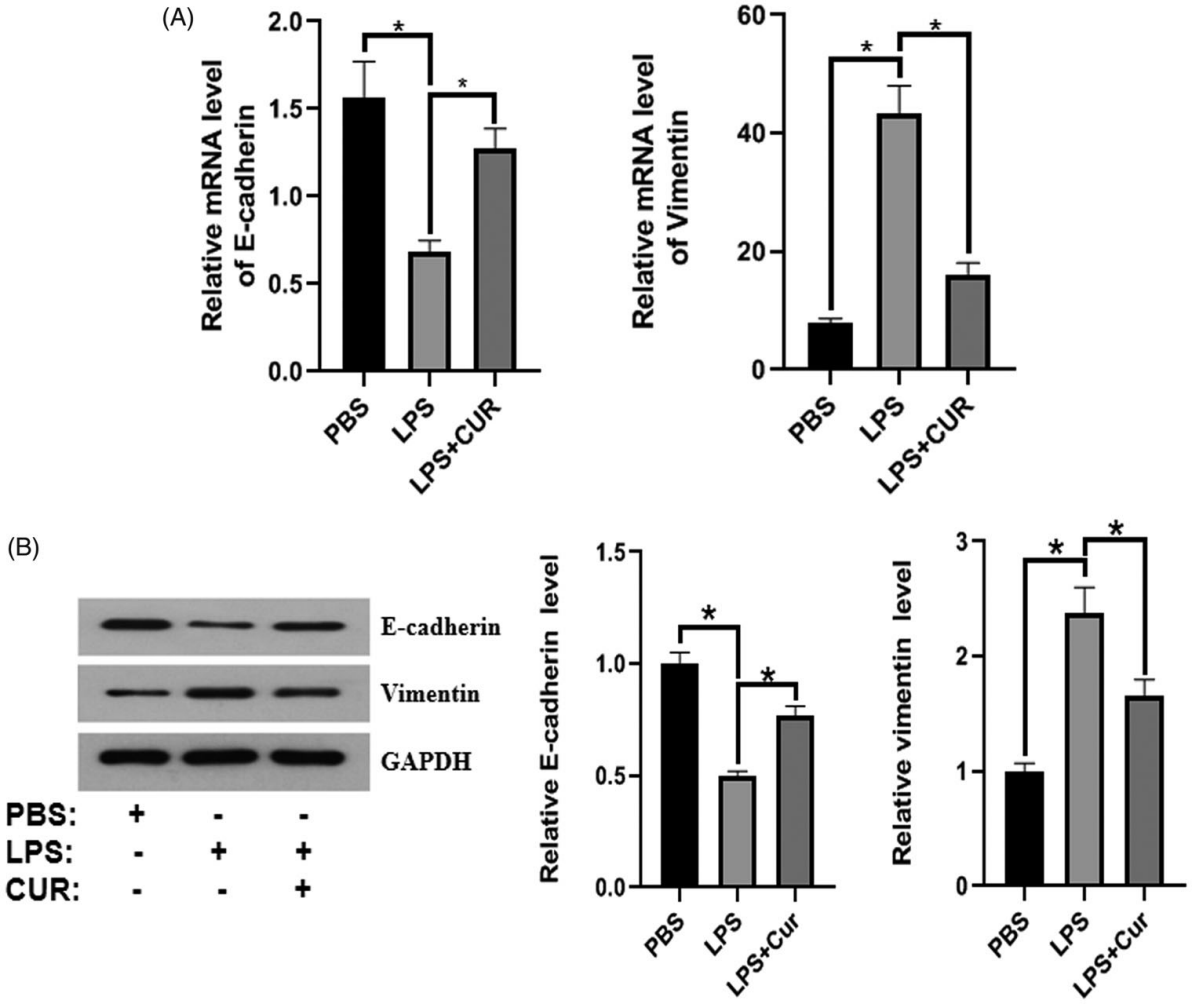
The effect of curcumin on the expression of E-cadherin and vimentin in the LPS-induced BPH mouse model (*n* = 8). (A,B) Protein and mRNA expression of E-cadherin and vimentin. *p < 0.05.

To explore the effect of curcumin on EMT *in vivo*, we first isolated primary prostate epithelial cells from the prostate tissue of mice (see details in the Materials and methods section). PCR and western blot analyses were performed to detect the expression of EMT markers. As illustrated in Figures 2(A,B), E-cadherin expression in the LPS group was lower than that in the control group, whereas treatment with curcumin significantly reversed the LPS-induced reduction. Furthermore, curcumin significantly promoted LPS-induced reduction in vimentin expression. Collectively, curcumin reduced EMT in the LPS-induced BPH mouse model by upregulating the expression of E-cadherin and downregulating that of vimentin.

### Effect of curcumin on the expression of BAMBI in the LPS-induced BPH mouse model

**Figure 3.**
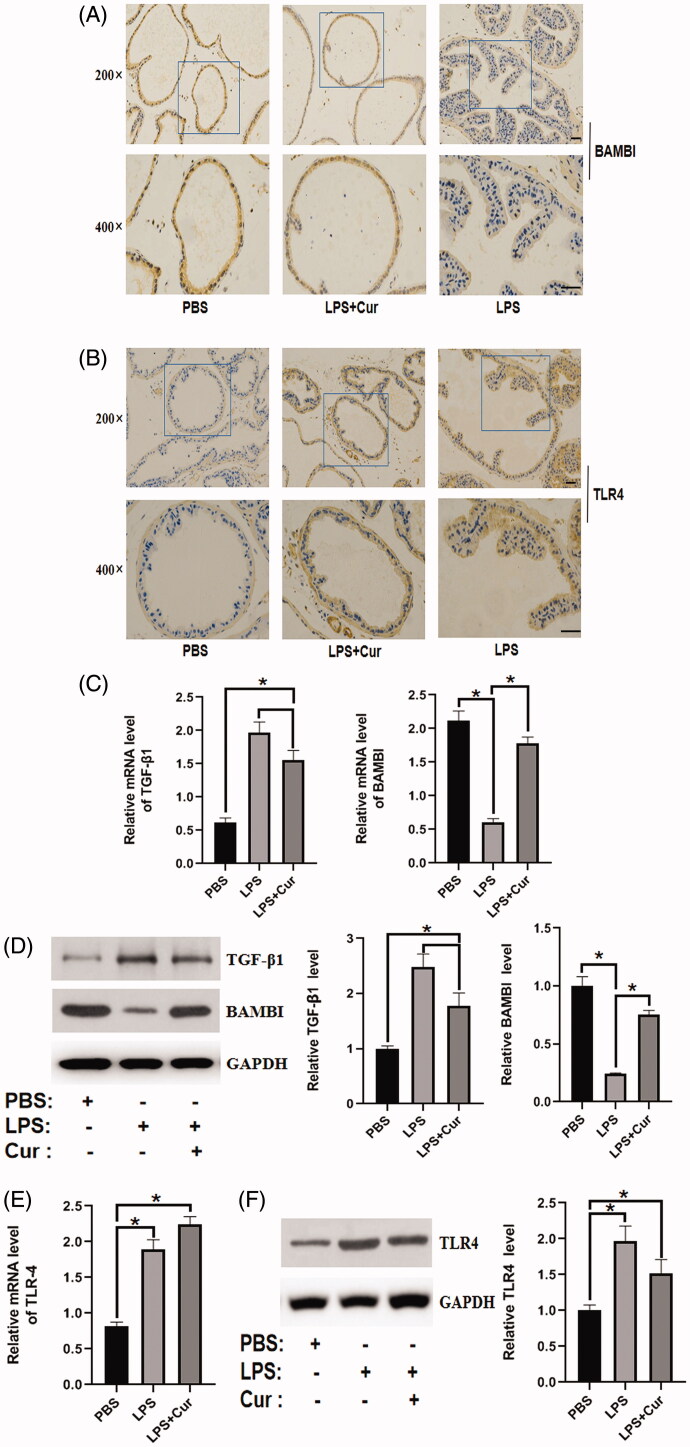
Effect of curcumin on the expression of BAMBI, TLR4, and TGF-β1 in the LPS-induced BPH mouse model (*n* = 8). (A) The expression of BAMBI in different groups, detected by immunohistochemistry (top panel: 200× magnification, bar = 50 μm; bottom panel: 400× magnification, bar = 50 μm). (B) The expression of TLR4 in various groups, as evidenced by immunohistochemistry (top panel: 200× magnification, bar = 50 μm; bottom panel: 400× magnification, bar = 50 μm). (C,D) mRNA and protein expression of BAMBI and TGF-β1 in each group, respectively (*n* = 8). (E,F) mRNA and protein expression level of TLR4 in each group. *p < 0.05.

The expression of BAMBI was notably downregulated in the LPS group compared to the control group, while the administration of curcumin dramatically reversed the reduction in BAMBI expression induced by LPS stimulation, as evidenced by immunohistochemistry, PCR, and western blotting (Figures 3(A,C,D)). Furthermore, the expression of TLR4 was higher in the LPS group than in the control group, and no significant difference in TLR4 expression was observed between the LPS group and the curcumin-treated group, as evidenced by immunohistochemistry, PCR, and western blotting (Figures 3(B,E)). Moreover, LPS stimulation dramatically increased the expression of TGF-β1 compared to that in the control group, while treatment with curcumin notably reversed the effect of LPS stimulation (Figures 3(C,D)). Accordingly, these results revealed that curcumin upregulated the expression of BAMBI in the LPS-induced BPH mouse model.

### Effect of curcumin on proliferation and EMT in BPH-1 cells

**Figure 4.**
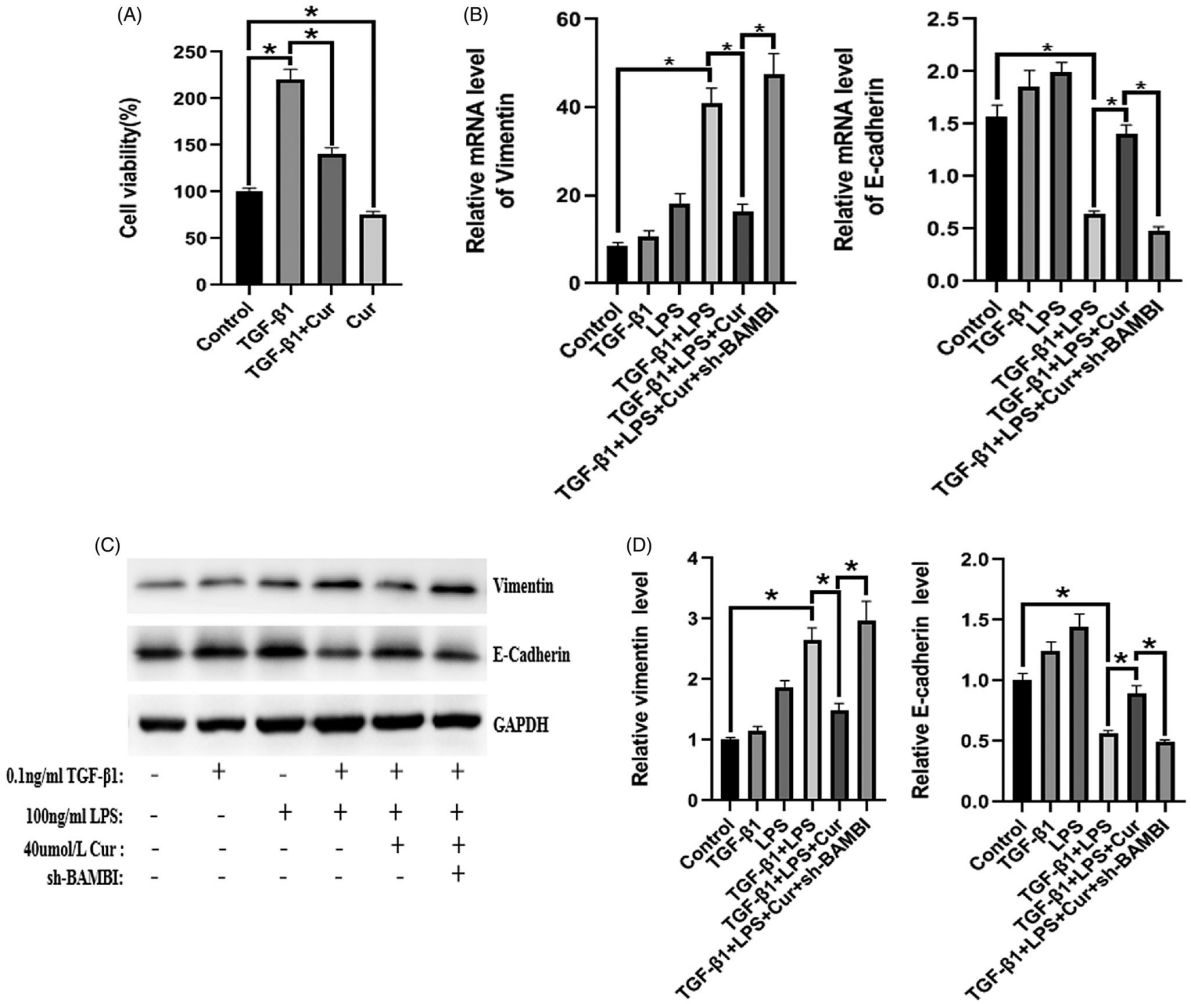
The effect of curcumin on the expression of E-cadherin and vimentin in BPH-1 cells. (*n* = 8). (A) Viability of BPH-1 cells under different treatments. (B–D) mRNA and protein expression of E-cadherin and vimentin in BPH-1 cells in each group, respectively. *p < 0.05.

As illustrated in Figure 4(A), the viability of BPH-1 cells in the experimental group was significantly decreased compared to that in the control group either in the presence or absence of TGF-β1 stimulation. In addition, combined treatment with LPS and TGF-β1 notably decreased the expression of vimentin and enhanced the expression of E-cadherin compared to the control group, while this tendency was markedly reversed by treatment with curcumin, indicating that curcumin can suppress EMT in LPS/TGF-β1-stimulated BPH-1 cells (Figures 4(B–D)). These data suggest that curcumin has an anti-proliferative effect and can significantly attenuate EMT induced by the combined stimulation of LPS and TGF-β1 in BPH-1 cells.

### EMT regulation by curcumin by targeting BAMBI via TLR4/BAMBI/TGF-β1 signalling pathway in BPH-1 cells

To confirm the mechanism of action of curcumin, we knocked down BAMBI in BPH-1 cells for further experiments. As illustrated in Figures 5(A–D), the expression of BAMBI in the LPS + TGF-β1 group was lower than that in the control group, whereas the administration of curcumin further facilitated the reduction in BPH-1 cells induced by the combined treatment of TGF-β1 and LPS according to the results of PCR, western blotting, and fluorescence immunohistochemistry. Furthermore, the BAMBI knockdown reversed the above-mentioned effect induced by curcumin. In addition, BAMBI knockdown reversed the attenuation of EMT by curcumin in TGF-β1/LPS-stimulated-BPH-1 cells (Figures 5(B–D)). Additionally, the expression of p-Smad2/3 in the LPS + TGF-β1 + Cur group notably decreased compared with that in the LPS + TGF-β1 group, while BAMBI knockdown reversed the above-mentioned downregulation by curcumin (Figure 5(C)). Moreover, LPS stimulation significantly increased the expression of TLR4 compared to the control group, while there was no significant difference in TLR4 expression between the LPS + TGF-β1 + Cur group and LPS + TGF-β1 group. To explore the interaction between TGF-β1 and BAMBI in cells, co-immunoprecipitation was performed. As shown in Figure 5(E), curcumin significantly downregulated the expression of the TGF-β1-BAMBI complex in cells, suggesting that curcumin achieves its anti-EMT effect by targeting BAMBI. Collectively, these data demonstrated that curcumin regulated EMT, probably by targeting BAMBI via the TLR4/BAMBI/TGF-β1 signalling pathway in BPH-1 cells.

Figure 5The effect of curcumin on the expression of BAMBI and TLR4 in BPH-1 cells (*n* = 8). (A) The expression of BAMBI and TLR4 in BPH-1 cells in each group, detected by immunofluorescence (BAMBI is shown in red, TLR4 is shown in green). (B–C) Relative mRNA expression of BAMBI and TLR4 in BPH-1 cells. (D) The protein expression of TLR4, p-Smad2/3, and BAMBI in BPH-1 cells in each group. (E) Expression of BAMBI and TGF-β1 immune complex in BPH-1 cells, as evidenced by Co-IP. *p < 0.05.

**Figure:**
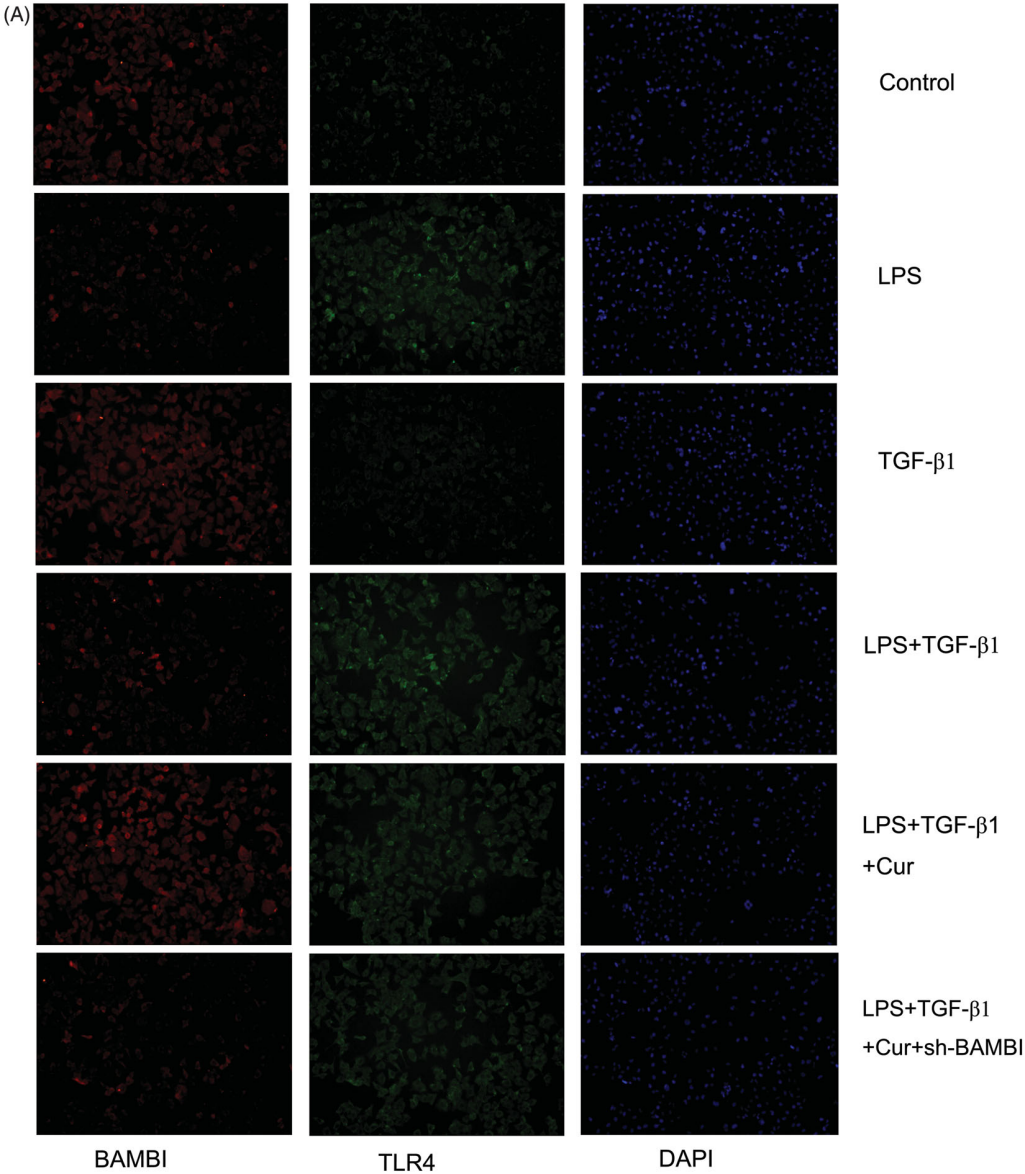

**Figure:**
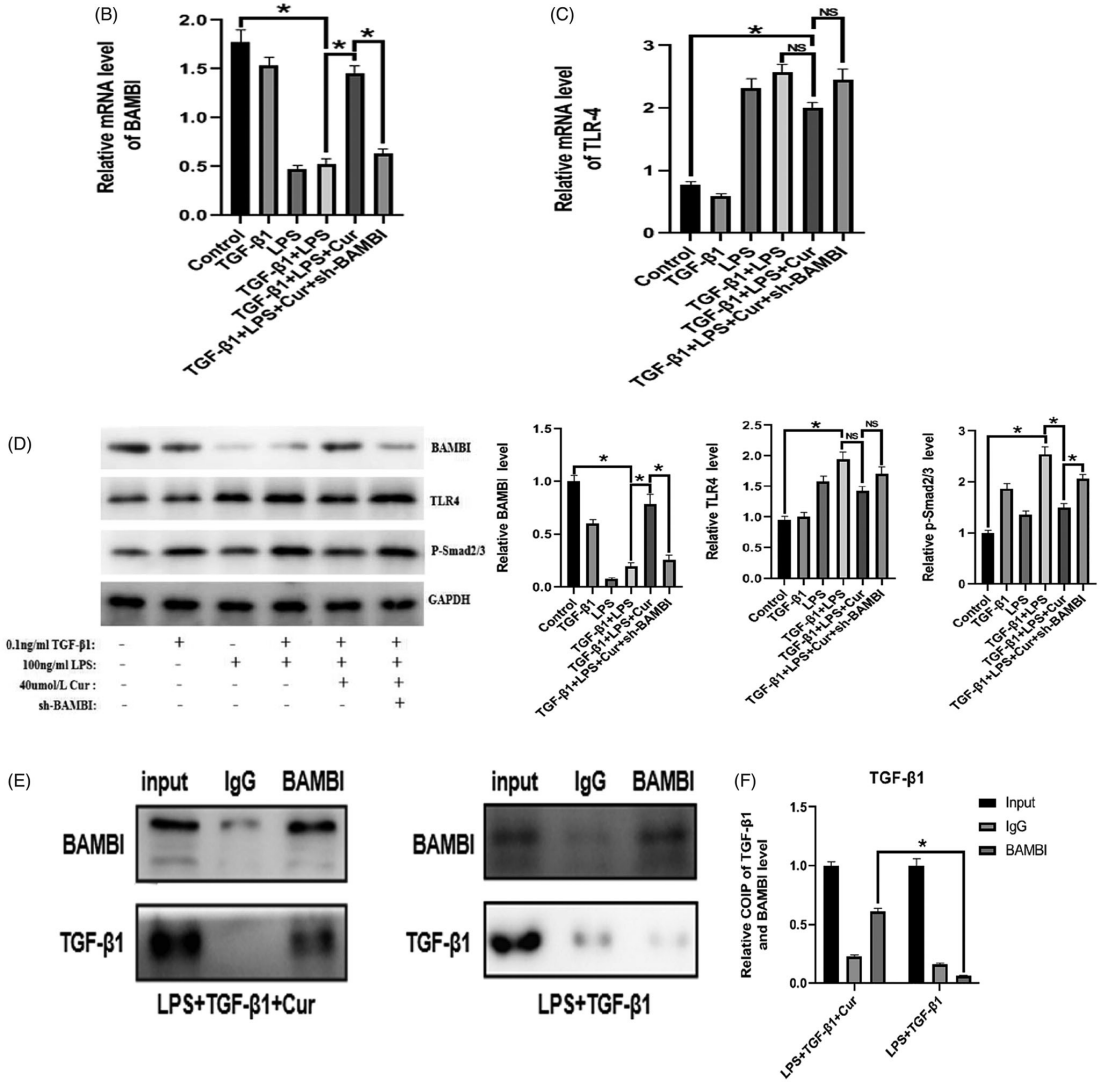

## Discussion

BPH is a benign disease that severely affects the quality of life of middle-aged and elderly men, and its pathogenic factors and treatment methods have attracted much attention (Speakman et al. 2015). Many studies have shown that there is a close relationship between prostatic inflammation and BPH (Robert et al. 2009; Zlotta et al. 2014). Currently, there is a lack of clinically effective anti-inflammatory drugs for the treatment of BPH. Furthermore, non-steroidal anti-inflammatory drugs (NSAIDs), which are commonly used clinically, have side effects that render them unsuitable for long-term use in patients with BPH (Geusens et al. 2013). To find new, safe, and effective anti-inflammatory drugs with minimal side effects, researchers have begun to focus on natural plant components that possess anti-inflammatory properties. Among them, curcumin, a plant extract, has been proven to have potent anti-inflammatory, anti-EMT, antitumor, and antifibrotic effects (Du et al. 2015; Kong et al. 2015; Derosa et al. 2016; Karimian et al. 2017; Bahrami et al. 2019). Therefore, its potential for clinical application in treating BPH has attracted much attention. Clinical trials have shown that curcumin can alleviate the inflammatory response with no side effects in patients with BPH who underwent transurethral resection of the prostate (Cosentino et al. 2016). In addition, considering that EMT is an inevitable process in the occurrence and development of BPH (Lu et al. 2012; Shao et al. 2014; Xu et al. 2019), we selected curcumin, a drug that has both anti-inflammatory and anti-EMT properties, for the present study.

Our previous study has shown that LPS is a key factor in the LPS/TLR4/BAMBI/TGF-β1 signalling pathway for low-dose TGF-β1 induction of EMT in BPH-1 cells (He et al. 2016). As an agonist of the TLR4 receptor, LPS can upregulate the expression of the TLR4 receptor (Gu et al. 2015). In addition, Wang et al. (2020) found that curcumin can downregulate the expression of TLR4 to inhibit EMT, thereby reducing renal interstitial fibrosis in obstructive nephropathy. Similarly, Atabaki et al. (2020) showed that curcumin could downregulate the expression of TLR4. In contrast, our research demonstrated that curcumin attenuated EMT in BPH-1 cells induced by LPS and TGF-β1, by upregulating BAMBI. Moreover, our study revealed that BAMBI is the target of curcumin in the LPS/TLR4/BAMBI signalling pathway. To further clarify the role of curcumin in the LPS/TLR4/BAMBI signalling pathway, we knocked down the expression of BAMBI in BPH-1 cells and observed that curcumin could not achieve its anti-EMT effect in BPH-1 cells. Similarly, our *in vivo* experiments also proved that the target of curcumin is BAMBI and not TLR4.

Hu et al. (2014) reported that TGF-β1 is an important factor that mediates EMT in BPH-1 cells. Our previous studies also indicated that high concentrations of TGF-β1 can directly promote EMT in BPH-1 cells via the TGF-β1/p-Smad2/3 signalling pathway. In our *in vitro* experiment, TGF-β1 was used to induce EMT in BPH-1 cells to explore the effect of curcumin on the LPS/TLR4/BAMBI signalling pathway. Previous studies have shown that curcumin can inhibit the expression of TGF-β1 in the myocardium and lung tissues (Kumari et al. 2017; Ma et al. 2017). Therefore, to investigate the effect of curcumin on TGF-β1 expression in prostate tissue *in vivo*, we measured the expression of TGF-β1 in the prostate tissue of each group of mice *in vivo*. Our results indicated that LPS could promote the expression of TGF-β1 in mouse prostate tissue, and curcumin reduced the expression of TGF-β1; however, the effect of curcumin was not significant. These results suggested that curcumin does not exert its anti-EMT effect by inhibiting the expression of TGF-β1 in mouse prostate tissue.

Kim et al. (2015) showed that curcumin can reduce testosterone-induced BPH in rats. Morphologically, curcumin can attenuate the proliferation of the epithelial cell layer and normalize the lumen space. In the present study, an LPS-induced BPH mouse model was used, which can induce prostatic hyperplasia along with an inflammatory response. Our results revealed that curcumin has an effect on reducing BPH as well as an anti-inflammatory effect. Curcumin could significantly reduce the infiltration of inflammatory cells into prostate tissues. Wang et al. (2020) indicated that curcumin could reduce the levels of IL-6, IL-1β, and TNF-α in UUO mice by 22.5, 30.3, and 26.7%, respectively. Nonn et al. (2007) showed that curcumin, resveratrol, and 6-gingerol could upregulate the expression of MKP5 in prostate tissue and reduce the expression of COX-2, IL-6, and IL-8 in prostate epithelial cells, thus indicating their anti-inflammatory effect. In the present study, we observed that curcumin reduced the expression of inflammatory factors IL-6 and TNF-α in prostate tissue. IL-6 can mediate the acute phase responses at the beginning of acute inflammation (Nguyen et al. 2014), and TNF-α can trigger the recruitment of inflammatory cells by stimulating the expression of pro-inflammatory genes (Apte and Voronov 2002). Therefore, we observed a reduction in inflammatory cell infiltration in the prostate tissue after administration of curcumin.

## Conclusions

We demonstrated that curcumin alleviated hyperplasia, EMT, and inflammation *in vivo*. In addition, curcumin suppressed EMT by targeting BAMBI via the TLR4/BAMBI/TGF-β1 signalling pathway *in vitro*. Consequently, we propose that curcumin may serve as a potential therapeutic agent in BPH treatment.
